# Giant mediastinal bronchial artery aneurysm mimicking mediastinal mass: A case report and brief review of the literature

**DOI:** 10.1016/j.radcr.2022.02.015

**Published:** 2022-03-03

**Authors:** Aneri B. Balar, Dhairya A. Lakhani, Daniel Martin, Kelly T. Smith, Cathy Kim

**Affiliations:** Department of Radiology, West Virginia University, Morgantown, WV, 26506, USA

**Keywords:** Bronchial artery aneurysm, Bronchial artery pseudoaneurysm, Mediastinal mass

## Abstract

Bronchial artery aneurysm and pseudoaneurysm is a rare but life-threatening diagnosis due to catastrophic complications from rupture. Prompt detection and management is key to prevent complications. CT angiogram and digital subtraction angiography are preferred diagnostic imaging modalities. Being very uncommon, these entities can be misdiagnosed as a nonspecific mediastinal soft tissue mass, which can lead to delay in diagnosis and inappropriate or delayed management. We present a case of 72-year-old woman with incidentally detected large bronchial artery pseudoaneurysm, incorrectly classified as mediastinal malignancy at outside facility, receiving follow-up exams for 2 years, before correct diagnosis and management.

## Introduction

Bronchial artery aneurysm is a rare finding, with less than 1% reported in patients undergoing selective bronchial arteriograms [1]. Bronchial artery pseudoaneurysm is extremely rare with few (less than 50) reported cases in the literature [1]. Bronchial artery pseudoaneurysm is usually asymptomatic, but can rupture and lead to catastrophic bleeding. Ruptured bronchial artery pseudoaneurysm may present with acute dyspnea, hemoptysis, hematemesis, hemothorax and hemorrhagic shock [1].

CT Angiography and digital subtraction angiography are diagnostic, with sensitivity of 67% and 100% respectively. These entities could be intrapulmonary, mediastinal or both. Bronchial artery pseudoaneurysm may be missed diagnostic on unenhanced CT as mediastinal lymphadenopathy and/or malignancy, delaying the diagnosis, as in our case [1,2]. Recognizing the pattern of presentation on unenhanced CT is critical to avoid fine-needle aspiration or core biopsy of such lesion which would lead to catastrophic bleeding [1].

Since bronchial artery pseudoaneurysm is life-threatening, prompt diagnosis and endovascular treatment regardless of presentation is advocated to present catastrophic complications from rupture. Management options include transcatheter embolization, covered stent placement, and surgical excision [1,[3], [4], [5]].

Here we present a case of a 72-year-old woman with incidentally detected large bronchial artery pseudoaneurysm, misdiagnosed as mediastinal lymphadenopathy on serial unenhanced cross-sectional imaging including CT and PET at outside facility.

## Case report

**Fig. 1:**
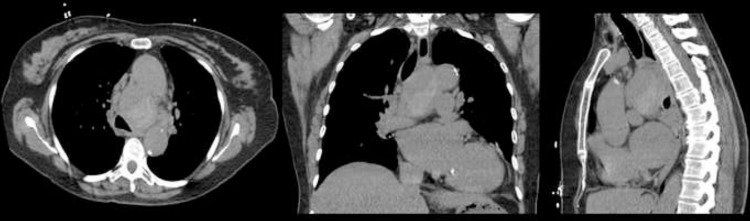
CT chest without contrast demonstrates a middle mediastinal, smooth, round mass measuring 4.2 x 5.5 x 6.7 cm, with internal areas of increased attenuation. This was reported as a soft tissue mass concerning for malignancy.

A 72-year-old female with no relevant past medical history presents to the clinic with chronic dyspnea. On arrival, she was afebrile and had stable vitals. CT chest without intravenous contrast was performed, which showed a middle mediastinal, smooth, round mass measuring 4.2 x 5.5 x 6.7 cm, with internal areas of increased attenuation (Fig. 1). Findings at outside facility were reported as concerning for malignancy.

**Fig. 2:**
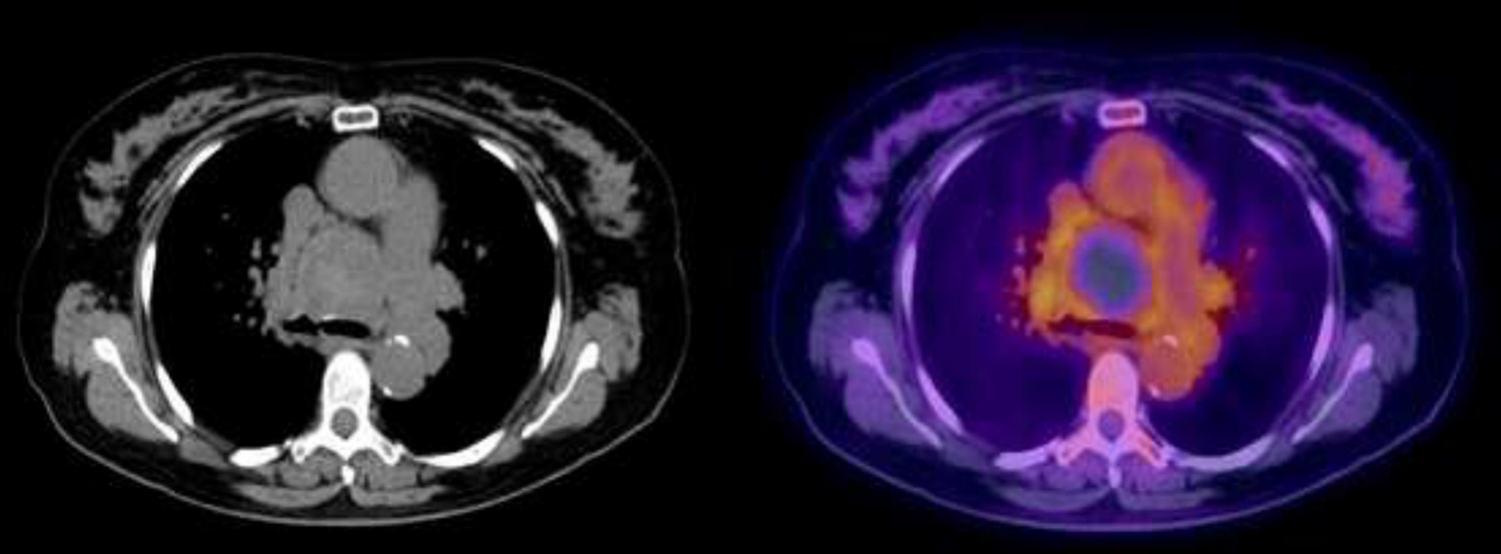
Subsequent analysis with unenhanced FDG PET/CT was performed. Increased metabolic activity as indicated by increased FDG uptake was noted in the periphery of the aforementioned mass, with central areas of no FDG uptake. This was reported as compatible with necrotizing mediastinal mass concerning for malignancy.

Subsequently, unenhanced FDG PET/CT was performed. Increased metabolic activity as indicated by increased FDG uptake was noted in the periphery of the aforementioned mass, with central areas of no FDG uptake. Findings at outside facility were reported as compatible with necrotizing mediastinal mass concerning for malignancy (Fig. 2).

**Fig. 3:**
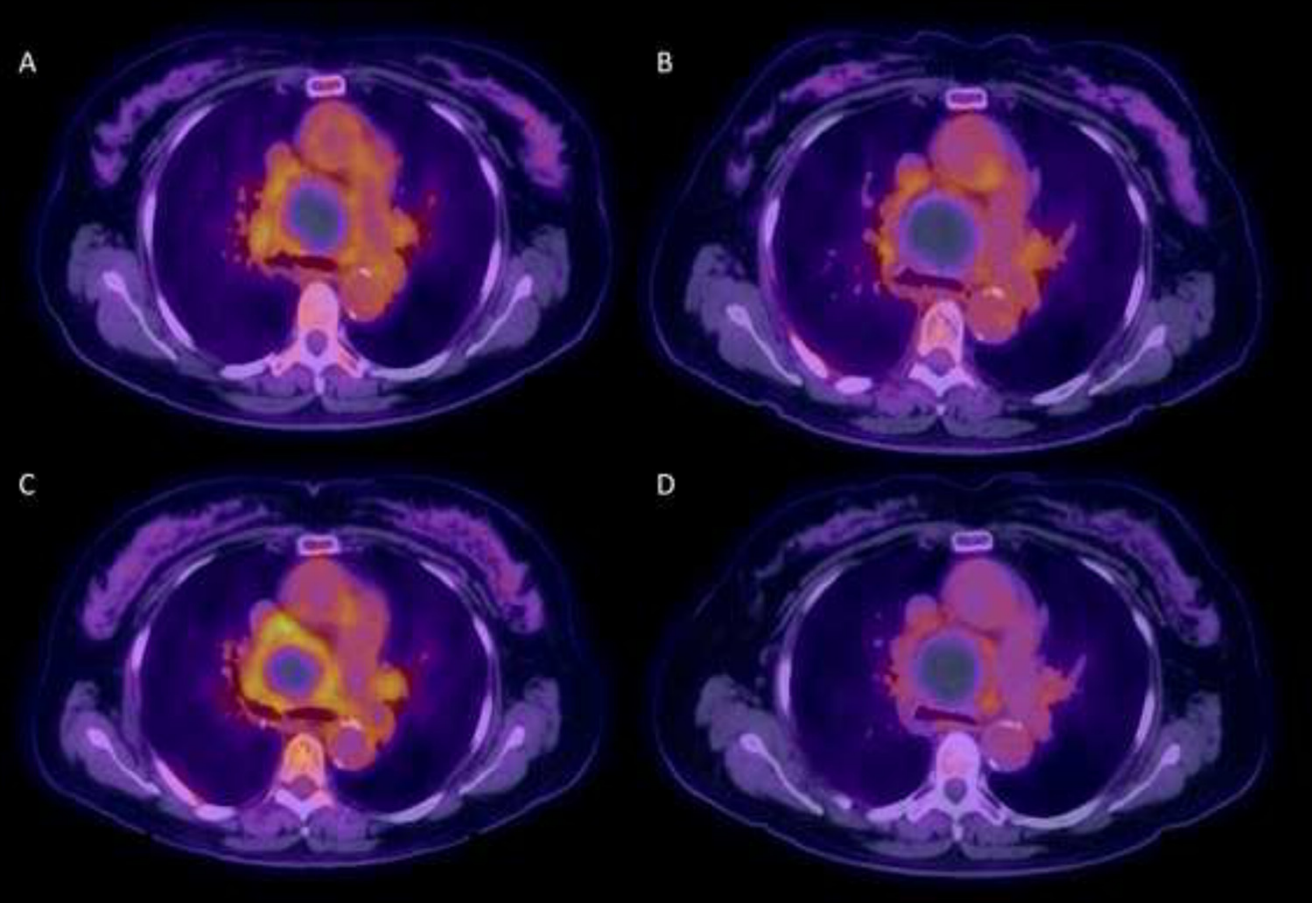
Serial follow-up unenhanced FDG PET/CT every 6-mo were performed for 2 y. (A) baseline, (B) 6-mo follow-up, (C) 12-mo follow-up, and (D) 24-mo follow-up. Findings were reported as unchanged from baseline exam at outside facility.

**Fig. 4:**
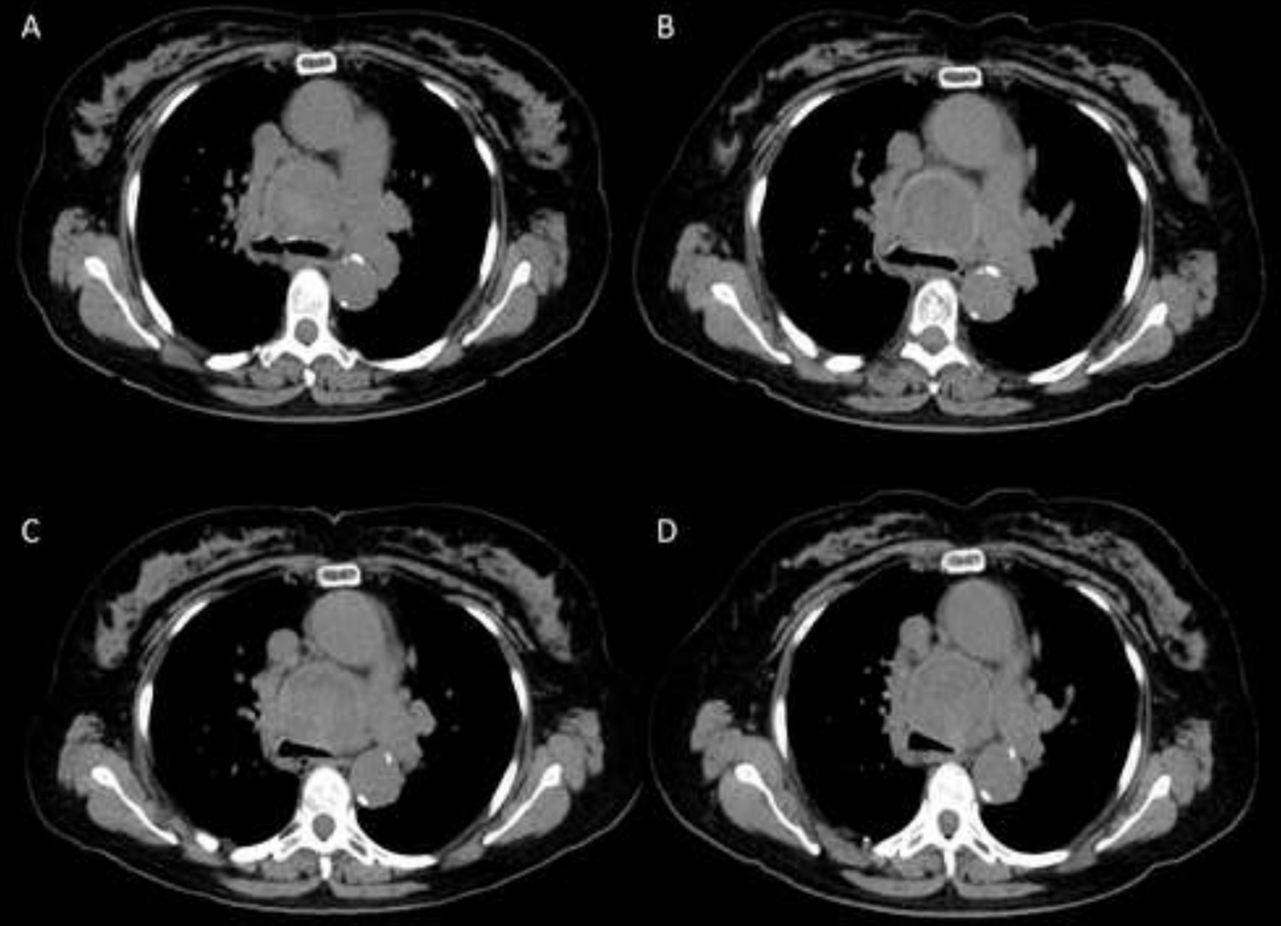
After receiving 2 y of serial imaging follow-up, patient was referred to our tertiary care center for further workup. Images from the outside institution were reviewed. (A) baseline, (B) 6-mo follow-up, (C) 12-mo follow-up, and (D) 24-mo follow-up. Examination of serial CT component of FDG PET/CT exams showed dynamic change in the internal hyperdense focus within the large round mediastinal mass throughout the examinations which was highly suggestive of a pseudoaneurysm as opposed to a malignant soft tissue mass. Further evaluation with CT angiogram was recommended.

**Fig. 5:**
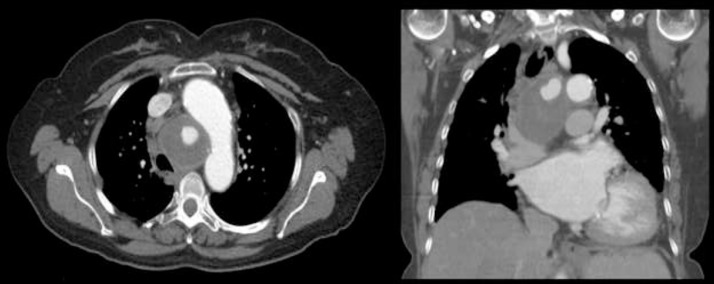
CT angiogram confirmed peripherally thrombosed bronchial artery pseudoaneurysm.

**Fig. 6:**
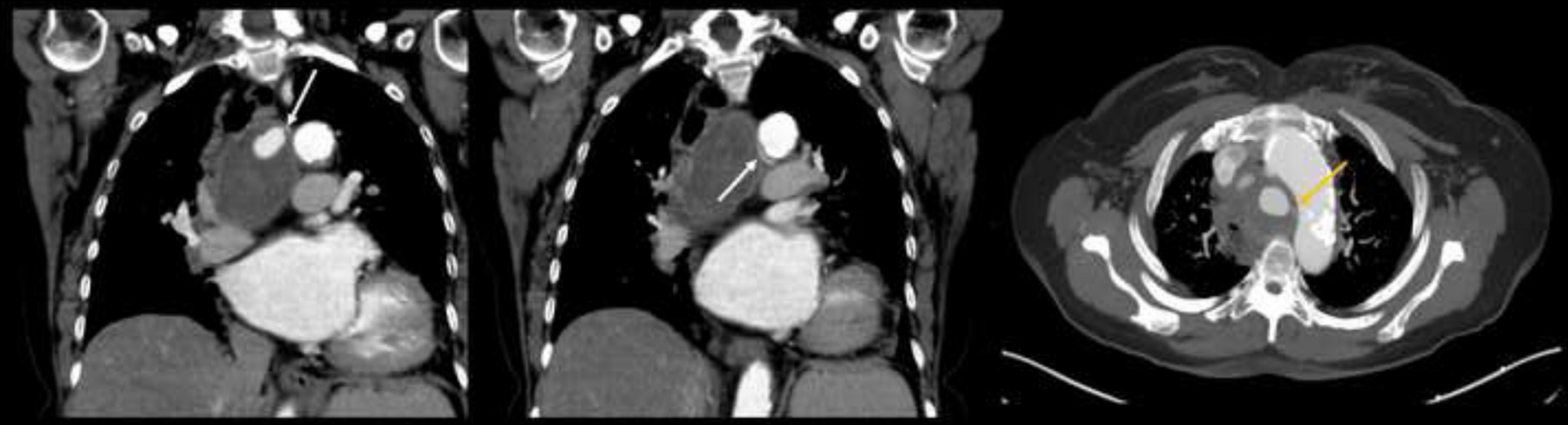
CT angiogram shows peripherally thrombosed pseudoaneurysm, arising from right bronchial artery (arrow).

Patient refused biopsy and hence received serial follow-up unenhanced FDG PET/CT every 6-month (Fig. 3), which were reported as stable findings by the reading radiologist. After receiving two-years of serial follow-up, patient was referred to our tertiary care center for further workup. Images from the outside institution were reviewed and examination of the serial CT component of FDG PET/CT exams (Fig. 4) showed dynamic change in the internal hyperdense focus within the large round mediastinal “mass”, which was highly suggestive of a pseudoaneurysm as opposed to malignancy. Further evaluation with contrast enhanced study (Fig. 5, Fig. 6) showed a peripherally thrombosed bronchial artery pseudoaneurysm.

**Fig. 7:**
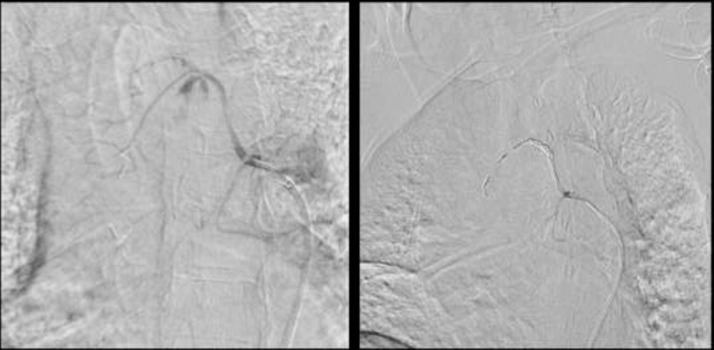
Representative images from selective right bronchial artery catheterization (using 5 Fr Cobra catheter) shows Pseudoaneurysm arising from midportion of superior branch of right bronchial artery. Subsequent coil embolization was performed.

Patient was subsequently treated with endovascular coil embolization (Fig. 7).

## Discussion

Etiology of bronchial artery aneurysm is not well understood. It can be congenital, related to pulmonary sequestration or pulmonary agenesis, or it can be acquired from atherosclerosis, inflammatory lung disease, bronchiectasis, infection or trauma [6], [7], [8], [9], [10], [11], [12], [13], [14].

Clinical presentation depends on the location of the lesion. Intrapulmonary bronchial artery aneurysm presents most commonly with hemoptysis. Whereas mediastinal bronchial artery aneurysm has more varied presentations including hemoptysis, dysphagia, dysphonia, hematemesis or hemoptysis if it ruptures into esophagus or airway [15].

Mediastinal bronchial artery aneurysm and pseudoaneurysm can be misdiagnosed as mediastinal soft tissue mass on imaging. There are several cases reported in the literature [2,6,11,[16], [17], [18], [19]]. Mediastinal lesions can be further stratified based on the location: anterior, middle, posterior and superior. Pathology in the mediastinum typically originate from lymph nodes, thymus, thyroid, esophagus, neurogenic, vascular and germ cell tumor [20]. Rare etiology would include amyloidosis or cardiac mass extending into the mediastinum [21], [22], [23], [24]. Intrapulmonary bronchial artery aneurysm presents with hemoptysis when ruptured into the trachea or airways. It can be misdiagnosed as a pulmonary nodule or mass [20].

Some important imaging features on unenhanced CT may help in prompt detection and correct characterization as aneurysm or hematoma, needing further assessment with contrast enhanced studies. These include: (1) Low-attenuation rounded structure adjacent to the vessels, and (2) Areas of intermediate or high attenuation (reflecting hemorrhage) adjacent to the above mentioned finding (pseudoaneurysm) indicating pseudoaneurysm rupture. The attenuation will vary depending on the chronicity and over time the internal characteristics will change [3]. Contrast-enhanced CT has a diagnostic appearance, with contrast opacification of the entire cavity, and in some cases, there will be “partial filling”, suggestive of peripheral thrombosis or “non-filling”, suggestive of complete thrombosis [3]. On ultrasound it has a characteristic “yin-yang” sign on color flow due to the turbulent forward and backward flow, and a "to and fro" pattern may be seen with spectral Doppler exam [3].

In conclusion, a rare case of asymptomatic large bronchial artery pseudoaneurysm is presented. It was misdiagnosed as a nonspecific mediastinal soft tissue mass on multiple consecutive unenhanced exams for two-years. Although contrast enhanced CT and digital subtraction angiography are preferred imaging modalities, we discuss characteristic findings of pseudoaneurysm on unenhanced CT which could raise suspicion and prompt additional evaluation with appropriate imaging. Bronchial artery aneurysm/pseudoaneurysm can lead to catastrophic bleeding if ruptured or biopsied in error and hence require prompt detection and management.

## Patient consent

Informed consent was obtained from the patient. No identifiable information is shared in current case report.

## Appendix Supplementary materials

Image, application 1
