# Supplementary material for: Giant mediastinal bronchial artery aneurysm mimicking mediastinal mass: A case report and brief review of the literature
Source: Radiol Case Rep. 2022 Mar 3;17(5):1496–501. doi: 10.1016/j.radcr.2022.02.015 (PMC8898754; doi:10.1016/j.radcr.2022.02.015)
Supplement: Supplementary file 1 [file mmc1.docx]

**TITLE:** Giant mediastinal bronchial artery aneurysm mimicking mediastinal mass: A case report and brief review of the literature

**ABSTRACT**

Bronchial artery aneurysm and pseudoaneurysm is a rare but life-threatening diagnosis due to catastrophic complications from rupture. Prompt detection and management is key to prevent complications. CT angiogram and digital subtraction angiography are preferred diagnostic imaging modalities. Being very uncommon, these entities can be misdiagnosed as a nonspecific mediastinal soft tissue mass, which can lead to delay in diagnosis and inappropriate or delayed management. We present a case of 72 year old woman with no prior lung conditions or trauma. She was found to have a large, smooth, hypoattenuating, round mass in the middle mediastinum, with findings reported as concerning for malignancy. Subsequent follow-up unenhanced CT exams were performed for two years, which revealed no change. At 2-year follow-up, a contrast-enhanced CT was performed, which correctly classified the mass as bronchial artery pseudoaneurysm as opposed to a mediastinal soft tissue mass. Patient subsequently underwent endovascular coiling.

**KEY WORDS:**

Bronchial artery aneurysm; Bronchial artery pseudoaneurysm; Mediastinal mass

**INTRODUCTION**

Bronchial artery aneurysm is a rare finding, with less than 1% reported in patients undergoing selective bronchial arteriograms (1). Bronchial artery pseudoaneurysm is extremely rare with few (less than 50) reported cases in the literature (1). Bronchial artery pseudoaneurysm is usually asymptomatic, but can rupture and lead to catastrophic bleeding. Ruptured bronchial artery pseudoaneurysm may present with acute dyspnea, hemoptysis, hematemesis, hemothorax and hemorrhagic shock (1).

CT Angiography and digital subtraction angiography are diagnostic, with sensitivity of 67% and 100% respectively. These entities could be intrapulmonary, mediastinal or both. Bronchial artery pseudoaneurysm may be missed diagnostic on unenhanced CT as mediastinal lymphadenopathy and/or malignancy, delaying the diagnosis, as in our case (1, 2). Recognizing the pattern of presentation on unenhanced CT is critical to avoid fine-needle aspiration or core biopsy of such lesion which would lead to catastrophic bleeding (1).

Since bronchial artery pseudoaneurysm is life-threatening, prompt diagnosis and endovascular treatment regardless of presentation is advocated to present catastrophic complications from rupture. Management options include transcatheter embolization, covered stent placement, and surgical excision (1, 3-5).

Here we present a case of a 72-year-old woman with incidentally detected large bronchial artery pseudoaneurysm, misdiagnosed as mediastinal lymphadenopathy on serial unenhanced cross-sectional imaging including CT and PET at outside facility, and receiving bronchoscopy guided fine needle aspiration (reported as non-diagnostic).

**CASE REPORT**

A 72-year-old female with no relevant past medical history presents to the clinic with chronic dyspnea. On arrival, she was afebrile and had stable vitals. CT chest without intravenous contrast was performed, which showed a middle mediastinal, smooth, round mass measuring 4.2 cm x 5.5 cm x 6.7 cm, with internal areas of increased attenuation (Figure 1). Findings at outside facility were reported as concerning for malignancy.

Subsequently, unenhanced FDG PET/CT was performed. Increased metabolic activity as indicated by increased FDG uptake was noted in the periphery of the aforementioned mass, with central areas of no FDG uptake. Findings at outside facility were reported as compatible with necrotizing mediastinal mass concerning for malignancy (Figure 2).

Patient refused biopsy and hence received serial follow-up unenhanced FDG PET/CT every six-month (Figure 3), which were reported as stable findings by the reading radiologist. After receiving two-years of serial follow-up, patient was referred to our tertiary care center for further workup. Images from the outside institution were reviewed and examination of the serial CT component of FDG PET/CT exams (Figure 4) showed dynamic change in the internal hyperdense focus within the large round mediastinal “mass”, which was highly suggestive of a pseudoaneurysm as opposed to malignancy. Further evaluation with contrast enhanced study (Figure 5 and 6) showed a peripherally thrombosed bronchial artery pseudoaneurysm.

Patient was subsequently treated with endovascular coil embolization (Figure 7).

**DISCUSSION**

Etiology of bronchial artery aneurysm is not well understood. It can be congenital, related to pulmonary sequestration or pulmonary agenesis, or it can be acquired from atherosclerosis, inflammatory lung disease, bronchiectasis, infection or trauma (6-14).

Clinical presentation depends on the location of the lesion. Intrapulmonary bronchial artery aneurysm presents most commonly with hemoptysis. Whereas mediastinal bronchial artery aneurysm has more varied presentations including hemoptysis, dysphagia, dysphonia, hematemesis or hemoptysis if it ruptures into esophagus or airway (15).

Mediastinal bronchial artery aneurysm and pseudoaneurysm can be misdiagnosed as mediastinal soft tissue mass on imaging. There are several cases reported in the literature (2, 6, 11, 16-19). Mediastinal lesions can be further stratified based on the location: anterior, middle, posterior and superior. Pathology in the mediastinum typically originate from lymph nodes, thymus, thyroid, esophagus, neurogenic, vascular and germ cell tumor (20). Rare etiology would include amyloidosis or cardiac mass extending into the mediastinum (21-24). Intrapulmonary bronchial artery aneurysm presents with hemoptysis when ruptured into the trachea or airways. It can be misdiagnosed as a pulmonary nodule or mass (20).

Some important imaging features on unenhanced CT may help in prompt detection and correct characterization as aneurysm or hematoma, needing further assessment with contrast enhanced studies. These include: 1) Low-attenuation rounded structure adjacent to the vessels, and 2) Areas of intermediate or high attenuation (reflecting hemorrhage) adjacent to the above mentioned finding (pseudoaneurysm) indicating pseudoaneurysm rupture. The attenuation will vary depending on the chronicity and over time the internal characteristics will change (3). Contrast-enhanced CT has a diagnostic appearance, with contrast opacification of the entire cavity, and in some cases there will be “partial filling”, suggestive of peripheral thrombosis or “non-filling”, suggestive of complete thrombosis (3). On ultrasound it has a characteristic “yin-yang” sign on color flow due to the turbulent forward and backward flow, and a "to and fro" pattern may be seen with spectral Doppler exam (3).

In conclusion, a rare case of asymptomatic large bronchial artery pseudoaneurysm is presented. It was misdiagnosed as a nonspecific mediastinal soft tissue mass on multiple consecutive unenhanced exams for two-years. Although contrast enhanced CT and digital subtraction angiography are preferred imaging modalities, we discuss characteristic findings of pseudoaneurysm on unenhanced CT which could raise suspicion and prompt additional evaluation with appropriate imaging. Bronchial artery aneurysm / pseudoaneurysm can lead to catastrophic bleeding if ruptured or biopsied in error and hence require prompt detection and management.

**REFERENCES:**

1. Kaufman C, Kabutey NK, Sgroi M, Kim D. Bronchial artery pseudoaneurysm with symptomatic mediastinal hematoma. Clin Imaging 2014;38(4):536-539. doi: 10.1016/j.clinimag.2014.01.010

2. Tanaka K, Ihaya A, Horiuci T, Morioka K, Kimura T, Uesaka T, Sasaki M, Uchinami M, Tsuda T, Yamada N, Li W, Hirai S, Tanabe S, Okubo Y, Tanaka S. Giant mediastinal bronchial artery aneurysm mimicking benign esophageal tumor: a case report and review of 26 cases from literature. J Vasc Surg 2003;38(5):1125-1129. doi: 10.1016/s0741-5214(03)00707-9

3. Saad NE, Saad WE, Davies MG, Waldman DL, Fultz PJ, Rubens DJ. Pseudoaneurysms and the role of minimally invasive techniques in their management. Radiographics 2005;25 Suppl 1:S173-189. doi: 10.1148/rg.25si055503

4. Zugazaga A, Stachno MA, García A, Tovar G, Benito V, Guasch I, Nogueira I, Puyalto P, Sampere J. Pulmonary artery pseudoaneurysms: endovascular management after adequate imaging diagnosis. Eur Radiol 2021;31(9):6480-6488. doi: 10.1007/s00330-021-07819-8

5. Urlings TAJ, Irani FG, Velaga J, Too CW. Ultrasound- and Fluoroscopic-Guided Embolization of a Bronchial Artery Pseudoaneurysm in a Patient with Lung Cancer. J Vasc Interv Radiol 2017;28(9):1323-1325. doi: 10.1016/j.jvir.2017.04.015

6. Abet D, Pietri J. [Ruptured bronchial artery aneurysm simulating dissection of the aorta in a patient with bronchopulmonary sequestration (author's transl)]. J Chir (Paris) 1981;118(12):743-746.

7. Lin J, Wood DE. Bronchial artery aneurysm refractory to transcatheter embolization. Ann Thorac Surg 2008;86(1):306-308. doi: 10.1016/j.athoracsur.2008.01.033

8. Misselt AJ, Krowka MJ, Misra S. Successful coil embolization of mediastinal bronchial artery aneurysm. J Vasc Interv Radiol 2010;21(2):295-296. doi: 10.1016/j.jvir.2009.10.030

9. Reade CC, Jenkins NL, Bard MR, Kuszyk BS, Koutlas TC, Rotondo MF. Immediate diagnosis and nonoperative treatment of a pulmonary artery pseudoaneurysm after blunt traumatic injury. J Trauma 2006;60(4):894-896. doi: 10.1097/01.ta.0000214598.48638.b5

10. Kalangos A, Khatchatourian G, Panos A, Faidutti B. Ruptured mediastinal bronchial artery aneurysm: a dilemma of diagnosis and therapeutic approach. J Thorac Cardiovasc Surg 1997;114(5):853-856. doi: 10.1016/S0022-5223(97)70094-1

11. Cearlock JR, Fontaine AB, Urbaneja A, Spigos DG. Endovascular treatment of a posttraumatic bronchial artery pseudoaneurysm. J Vasc Interv Radiol 1995;6(3):495-496. doi: 10.1016/s1051-0443(95)72852-4

12. You CK, Whatley GS. Swan-Ganz catheter-induced pulmonary artery pseudoaneurysm: a case of complete resolution without intervention. Can J Surg 1994;37(5):420-424.

13. Dallaudière B, Hummel V, Pierre Laissy J. The use of covered stents for treatment of pulmonary artery pseudoaneurysms. J Vasc Interv Radiol 2013;24(2):296-298. doi: 10.1016/j.jvir.2012.10.024

14. Ghonge NP. Bronchial Artery Pseudoaneurysm from a Tuberculous Lymph Node. Radiology 2021;300(1):37. doi: 10.1148/radiol.2021204090

15. Jadeja S, Green K, Shuja A, Malespin M, De Melo S. Esophageal Bronchial Artery Fistulaization: A Complication of an Endobronchial Ultrasound. ACG Case Rep J 2020;7(4):e00355. doi: 10.14309/crj.0000000000000355

16. Lioulias A, Misthos P, Kokotsakis J, Papagiannakis G, Skouteli E. Recurrent massive hemoptysis due to postbronchotomy bronchial artery aneurysm: a case report. Can Respir J 2008;15(3):127-128. doi: 10.1155/2008/712302

17. Chantepie A, Robert M, Pelletier J, Gold F, Mercier C, Lacombe A, Laugier J. [Mycotic aneurysm of bronchial artery. Apropos of a case in an infant]. Chir Pediatr 1980;21(6):407-410.

18. Watanabe Y. [Bronchial artery aneurysm]. Ryoikibetsu Shokogun Shirizu 1994(3):627-628.

19. Remy-Jardin M, Remy J, Ramon P, Fellous G. Mediastinal bronchial artery aneurysm: dynamic computed tomography appearance. Cardiovasc Intervent Radiol 1991;14(2):118-120. doi: 10.1007/BF02577709

20. Whitten CR, Khan S, Munneke GJ, Grubnic S. A diagnostic approach to mediastinal abnormalities. Radiographics 2007;27(3):657-671. doi: 10.1148/rg.273065136

21. Crain MA, Lakhani DA, Balar AB, Hogg JP, Adelanwa A, Hailemichael E. Tracheobronchial amyloidosis: A case report and review of literature. Radiol Case Rep 2021;16(9):2399-2403. doi: 10.1016/j.radcr.2021.05.082

22. Crain MA, Vasilakis GM, Adkins JR, Adelanwa A, Hogg JP, Lakhani DA, Kim C. Primary nodular chest amyloidoma: A case report and review of literature. Radiol Case Rep 2022;17(3):631-637. doi: 10.1016/j.radcr.2021.11.048

23. Lakhani DA, Balar AB, Kim C. Prominent crista terminalis mimicking a right atrial mass: A case report and brief review of the literature. Radiol Case Rep 2022;17(3):434-438. doi: 10.1016/j.radcr.2021.11.028

24. Vasilakis GM, Lakhani DA, Adelanwa A, Hogg JP, Kim C. Atypical imaging presentation of a massive intracavitary cardiac thrombus: A case report and brief review of the literature. Radiol Case Rep 2021;16(10):2847-2852. doi: 10.1016/j.radcr.2021.06.089

**FIGURE LEGENDS:**

**Figure 1:** CT chest without contrast demonstrates a middle mediastinal, smooth, round mass measuring 4.2 cm x 5.5 cm x 6.7 cm, with internal areas of increased attenuation. This was reported as a soft tissue mass concerning for malignancy.

**Figure 2:** Subsequent analysis with unenhanced FDG PET/CT was performed. Increased metabolic activity as indicated by increased FDG uptake was noted in the periphery of the aforementioned mass, with central areas of no FDG uptake. This was reported as compatible with necrotizing mediastinal mass concerning for malignancy.

**Figure 3:** Serial follow-up unenhanced FDG PET/CT every six-month were performed for two years. A) baseline, B) 6-month follow-up, C) 12-month follow-up, and D) 24-month follow-up. Findings were reported as unchanged from baseline exam at outside facility.

**Figure 4:** After receiving two years of serial imaging follow-up, patient was referred to our tertiary care center for further workup. Images from the outside institution were reviewed. A) baseline, B) 6-month follow-up, C) 12-month follow-up, and D) 24-month follow-up. Examination of serial CT component of FDG PET/CT exams showed dynamic change in the internal hyperdense focus within the large round mediastinal mass throughout the examinations which was highly suggestive of a pseudoaneurysm as opposed to a malignant soft tissue mass. Further evaluation with CT angiogram was recommended.

**Figure 5:** CT angiogram confirmed peripherally thrombosed bronchial artery pseudoaneurysm.

**Figure 6:** CT angiogram shows peripherally thrombosed pseudoaneurysm, arising from right bronchial artery (arrow).

**Figure 7:** Representative images from selective right bronchial artery catheterization (using 5 Fr Cobra catheter) shows Pseudoaneurysm arising from midportion of superior branch of right bronchial artery. Subsequent coil embolization was performed.
